# What can be done to reduce surgical waiting time for pediatric ent in public health care in Brazil?

**DOI:** 10.1016/j.bjorl.2025.101700

**Published:** 2025-08-22

**Authors:** Reginaldo Raimundo Fujita, Shirley Shizue Nagata Pignatari, Vitor Guo Chen, Paula Angelica Lorenzon, Luciano Rodrigues Neves, Gabriela Ricci Lima Luz Matsumoto, Manoel de Nobrega, Ektor Tsuneo Onishi, Silvana Bommarito, Jaquelina Ota Araki

**Affiliations:** a Universidade Federal de São Paulo (UNIFESP), Escola Paulista de Medicina(EPM), Divisão de Otorrinolaringologia Pediátrica, São Paulo, SP, Brazil; b Universidade Federal de São Paulo (UNIFESP), Escola Paulista de Medicina(EPM), Departamento de Fonoaudiologia, São Paulo, SP, Brazil; c Universidade Federal de São Paulo (UNIFESP), Escola Paulista de Medicina(EPM), Divisão de Pneumologia, São Paulo, SP, Brazil

Waiting times for elective surgical procedures in Brazil’s public health system (Sistema Único de Saúde – SUS) have increased significantly, particularly following the onset of the SARS-CoV-2 pandemic. Between 2020 and 2021, the number of elective surgeries dropped by more than 30% across all regions of the country, resulting in an accumulated demand that has not yet been fully addressed.^1^ This trend is multifactorial and involves chronic underfunding, low reimbursement rates to service providers, prioritization of high-risk and emergency cases, and recurrent shortages of materials and surgical infrastructure.

Among the most affected are pediatric patients requiring low-complexity surgeries such as adenotonsillectomies and tympanostomy tube placements. Despite their relatively straightforward nature, these procedures are often prioritized, even though the benefits of early intervention are well established.^5^ The timely treatment of obstructive sleep disorders and restoration of hearing in children is strongly associated with improved physical growth, neurocognitive development, and social integration. Moreover, such interventions may reduce the need for pharmacological treatment, lower school failure rates, and alleviate the burden on primary care services.^2^^,^^3^

In response to this pressing public health issue, our team developed and implemented a targeted, multidisciplinary task force designed to reduce surgical backlogs among pediatric patients. This model has been in operation since 2023 at a tertiary university hospital and has produced encouraging outcomes. The initiative was conceived not only to address delays in care but also to serve as a platform for education, research, and community engagement.

The key components of the task force model include:

1 Weekend Scheduling: Elective pediatric surgeries are conducted on Saturdays, minimizing competition with higher-complexity cases performed on weekdays. This strategy optimizes operating room usage without disrupting routine hospital workflows.
2 Multidisciplinary Teamwork: Procedures are carried out by teams of otolaryngologists, anesthesiologists, audiologists, nurses, pediatricians, and nutritionists. The team composition ensures comprehensive perioperative care and fosters interprofessional collaboration.
3 Academic Integration: The program is aligned with institutional extension courses and includes structured participation from undergraduate students in medicine, nursing, and speech-language pathology. Activities are scheduled on weekends to avoid interference with curricular obligations, and certificates are issued to formally recognize student engagement.
4 Strategic Partnerships: Collaboration with private hospitals facilitates the loan of sterilized, ready-to-use surgical instruments, reducing the turnover time between cases. Medical supply companies contribute by donating consumables, such as sutures and ventilation tubes. These partnerships not only reduce costs but also encourage private sector involvement in public health initiatives.
5 Patient Safety and Selection: Candidates for surgery are carefully screened to exclude those with complex comorbidities, ensuring procedural safety and efficiency. A simplified protocol allows for streamlined triage, pre-anesthetic assessment, and hospital discharge.
6 Community Participation: Volunteer medical students assist in patient reception and the distribution of comfort items for children. This element enhances the humanization of care and strengthens the link between the academic institution and the surrounding population.

From an educational standpoint, the initiative offers a rich environment for experiential learning. First- and second-year medical students participate in the reception and orientation of patients and their families, promoting early exposure to patient-centered care. Third-year students conduct supervised preoperative evaluations, improving their clinical reasoning and familiarity with pediatric workflows.

More senior students — those in their fourth to sixth years — are integrated into the surgical and anesthetic teams as scrub assistants or anesthesiology aides. This immersive experience exposes them to surgical procedures, team-based care, and interprofessional communication within a real-world clinical context. The teaching value is amplified by the structured feedback and supervision provided by the faculty members in each discipline.

Over the past two years, we have conducted ten of these surgical campaigns, resulting for treating 200 pediatric patients who were previously on the waiting list. While this number may appear modest in isolation, it represents a significant local impact and a scalable model. Moreover, recent increases in SUS reimbursement rates for certain pediatric otolaryngological procedures have improved the cost-effectiveness of these initiatives, making them more attractive for replication across the country.^4^

Beyond the numerical outcomes, the social and educational benefits of the program deserve equal emphasis. Families reported increased satisfaction with the care process, and students consistently describe the experience as formative. The initiative aligns with broader health equity goals by providing timely care to underserved populations and integrating service delivery with academic training.

This model also responds to a critical need identified in the literature: the development of agile, community-based strategies to address surgical delays in resource-constrained settings^1^. By leveraging institutional infrastructure, academic engagement, and external partnerships, this task force demonstrates that innovation in public health does not always require new technologies or large investments - rather, it can emerge from coordinated, purposeful action.

We hope that our experience inspires other academic centers and public hospitals to adopt similar approaches. The reduction of surgical waiting lists, particularly for children, must be a national priority. As emphasized by Oliveira^1^, targeted interventions such as surgical task forces offer a viable, evidence-based solution to one of the most persistent challenges in the Brazilian health system.

## ORCID ID

Reginaldo Raimundo Fujita: 0000-0002-0222-3596

Shirley Shizue Nagata Pignatari: 0000-0002-4299-2226

Vitor Guo Chen: 0000-0002-2530-9093

Paula Angelica Lorenzon: 0009-0000-9529-4379

Luciano Rodrigues Neves: 0000-0001-6985-183X

Gabriela Ricci Lima Luz Matsumoto: 0000-0003-0793-153X

Manoel de Nobrega: 0000-0002-1342-633X

Ektor Tsuneo Onishi: 0000-0003-1501-8409

Silvana Bommarito: 0000-0003-3708-7878

Jaquelina Ota Araki: 0000-0002-5350-7679

## Declaration of competing interest

The authors declare no conflicts of interest.
